# Network hub changes in the pars opercularis indicate impaired inhibition in tic disorder patients

**DOI:** 10.1017/neu.2024.53

**Published:** 2025-01-27

**Authors:** SuHyuk Chi, Young Eun Mok, June Kang, Jeong-An Gim, Moon-Soo Lee

**Affiliations:** 1 Department of Psychiatry, Korea University Guro Hospital, Seoul, Korea; 2 Department of Brain and Cognitive Engineering, Korea University, Seoul, Korea; 3 Department of Medical Science, Soonchunhyang University, Asan-si, Chungcheongnam-do, Korea

**Keywords:** Tic disorders, tourette Syndrome, magnetic resonance imaging, brain, brain mapping

## Abstract

**Objective:**

This study aimed to utilise graph theory to explore the functional brain networks in individuals with tic disorders and to investigate resting-state functional connectivity changes in critical brain regions associated with tic disorders.

**Methods:**

Participants comprised individuals with tic disorders and age-matched healthy controls, ranging from 6 to 18 years old, all recruited from Korea University Guro Hospital. We ensured a medication-naïve cohort by excluding participants exposed to psychotropic medications for at least three weeks prior to the study. Data included structural and resting-state functional MRI scans, analysed with the CONN-fMRI Functional Connectivity toolbox v20b. The analysis included 22 patients (18 males, 4 females) and 26 controls (14 males, 12 females).

**Results:**

Significantly increased global efficiency was observed in the left inferior frontal gyrus pars opercularis among tic disorder patients compared to controls. Furthermore, this region displayed enhanced resting-state functional connectivity with its right counterpart in patients versus controls.

**Conclusion:**

The inferior frontal gyrus pars opercularis, known for its inhibitory role, may reflect adaptive functional adjustments in response to tic symptoms. Increased hubness of the inferior frontal gyrus pars opercularis possibly represents functional adjustments in response to tic symptoms. The identified brain region with increased efficiency and connectivity presents a promising avenue for further research into tic expression and control mechanisms.

Significant outcomes

- The left inferior frontal gyrus pars opercularis of tic disorder patients shows increased global efficiency compared to controls.
- The left inferior frontal gyrus pars opercularis shows enhanced resting-state functional connectivity with its right counterpart in patients compared to controls.

Limitations

- The small number of enrolled patients is a limitation of this study.
- The cross-sectional nature of our study precluded the verification of our results over a prospective period.

## Introduction

Tic disorders typically manifest at an early age and involve repetitive, involuntary movements or vocal sounds. They are notable prevalent with an incidence rate as high as 5.6% (Knight *et al*., 2012). The most severe manifestation of tic disorders is called Tourette syndrome, characterised by the concurrence of motor and vocal tics for over one year. While not life-threatening, tic disorders are associated with impaired self-esteem, social life, and academic performance. These issues may persist into adulthood, adversely influencing career choices and interpersonal relationships. Tic disorders are also linked to a higher prevalence of attention-deficit hyperactivity disorder (ADHD), which can independently lead to various psychiatric and behavioural problems. These challenges significantly impact not only the patients but often their entire families.

Our current understanding of tic disorders, derived from various imaging, genetic, and postmortem studies, suggests that tic disorders are associated with abnormalities in the corticospinal tract, particularly within the striatum. Imaging studies documented decreases in volume in the striatum and the globus pallidus in patients with tic disorders (Makki *et al*., 2009; Y. Worbe *et al*., 2015). Intriguingly, an ongoing study on tic disorder patients reported that the severity of the condition was predicted not by striatal volumes but by hippocampal volumes (Black *et al*., 2020). A relatively recent study proposed a model dividing the underlying mechanism into two discrete networks: the expression network and the control network (Yael *et al*., 2015). The expression network, associated with behavioural symptoms, includes motor-related areas such as the pre- and primary motor cortices, putamen, and limbic areas, as highlighted by functional magnetic resonance imaging (fMRI) studies (Neuner *et al*., 2014). Conversely, the control network, which modulates the expression of these behavioural symptoms, is thought to encompass the frontal cortical areas. This hypothesis is supported by a study showing correlations between tic suppression and increased regional homogeneity of the left inferior frontal gyrus (Ganos *et al*., 2014). Premonitory urges, on the other hand, are believed to span both networks, mediated by sensory and limbic brain regions (Neuner *et al*., 2014; Z. Wang *et al*., 2011). Church *et al.* also investigated the resting-state functional connections in 33 adolescents with tic disorder and 210 healthy individuals and found that patients showed many less mature functional connections in the fronto-parietal network and the cingulo-operculo network (Church *et al*., 2009). The authors defined ‘maturity’ as how developed each functional brain connection is in the patient group compared to typical development in people without the disease. Such defects in brain maturation was further supported by a study on the functional connectivity of 59 adult Tourette syndrome patients and 27 controls, revealing stronger functional integration and global disorganisation in sensory motor, associative, and limbic cortico-basal ganglia networks among patients (Yulia Worbe *et al*., 2012).

Our research endeavoured to extend these earlier studies by employing graph theory to conceptualise the brain as a network of interconnected nodes. Investigations on animal brains deducted that nervous systems possess properties of small-world networks, making them suitable for analysis using graph theory (Watts and Strogatz, 1998; Hilgetag *et al*., 2000). The advent of fMRI facilitated practical applications of graph theoretical analysis on the human brain. Early studies already reported small-world structures within cortical and subcortical regions of the brain network (Salvador *et al*., 2005). This methodology has been adopted in various clinical studies, exploring a range of psychiatric disorders, such as depression and schizophrenia, in greater depth (Su *et al*., 2015; Ye *et al*., 2015). The application of network analysis offers a comprehensive perspective on the brain’s connectivity, capturing both anatomical pathways and correlations in activity, providing a more nuanced understanding of whole-brain effective connectivity and the inherent dynamics of brain activity (Gilson *et al*., 2019; J. Wang *et al*., 2010). This was applied in tic disorder research by Wen et al., who constructed whole-brain, region of interest (ROI)-level functional connectivity networks for 29 drug-naive tic disorder children and 37 healthy controls, and used graph theory to investigate topological disruptions (Wen *et al*., 2018). The identified regions involved the sensorimotor, visual, language, and default-mode areas. They also analysed white matter networks in 44 tic disorder children and 41 healthy controls, finding that patients exhibited decreased global and local efficiency, increased path length, and disrupted network balance (Wen *et al*., 2017).

Despite this extensive amount of existing research, a comprehensive understanding of this complex disorder remains elusive. This study aimed to investigate the resting-state functional neuroimaging data of tic disorder patients through a data-driven network analysis followed by a hypothesis-driven functional connectivity analysis of the prior results.

## Material and methods

### Participants and clinical measures

A total of 39 patients with tic disorder and 37 healthy controls were initially enrolled in the study. All participants were between the ages of 6 and 18, psychotropic medication free for at least 3 weeks, and had no history of neurologic disorders including head trauma, tumours, or seizures. Patients were clinically diagnosed with tic disorders (Tourette’s disorder, persistent motor or vocal tic disorder, provisional tic disorder, other specified tic disorder, and unspecified tic disorder) based on the 5^th^ edition of the Diagnostic and Statistical Manual of Mental Disorder (DSM-5) by child and adolescent psychiatrists. Intelligence quotients of all patients and controls were examined using the Korean version of the Wechsler Intelligence Scale for Children fourth edition (K-WISC-IV). Patients were recruited from the department of psychiatry of Korea University Guro Hospital. Healthy controls were recruited from local schools and kindergartens.

Patients were assessed using the Korean version of the Kiddie-Schedule for Affective Disorders and Schizophrenia-Present and Lifetime Version (K-SADS-PL) for psychiatric comorbidities, and the Yale Global Tic Severity Scale (YGTSS) for tic disorder symptom severity.

The research processes were approved by the Institutional Review Board (IRB) of the Korea University Guro Hospital (2021GR0275). All research methods were performed in accordance with the relevant guidelines and regulations. Written consent was obtained from parents or legal guardians of each participant.

### Functional MRI image acquisition

Participants were instructed to close their eyes but stay awake, relax, and remain motionless throughout the scan. The head was stabilised using sponges, and sedatives were administered in case of excessive movement. A 3.0-Tesla Siemens MR scanner (MAGNETOM Prisma; SIEMENS Healthineers, Erlangen, Germany) at Korea University Guro Hospital was used. Structural data were scanned using a T1-weighted magnetisation-prepared rapid gradient-echo sequence (repetition time: 2,300 ms; echo time: 2.32 ms; inversion time: 900 ms; flip angle: 8°; field of view: 230 mm; voxel size: 0.9 mm isotropic; 208 slices; generalised auto-calibrating partially parallel acquisition acceleration factor: factor of two along the phase-encoding direction; received bandwidth per pixel: 200 Hz/pixel; echo spacing: 7.1 ms). Resting-state fMRI scans were acquired through 200 contiguous echo-planar imaging volumes of the whole brain (repetition time: 2,000 ms; echo time: 30 ms; flip angle: 70°; field of view: 224 mm; voxel size: 2.0 mm isotropic; multi-slice mode; interleaved; simultaneous multi-slice factor: three; phase-encoding shift factor: two; received bandwidth per pixel: 2,480 Hz/pixel; echo spacing: 0.55 ms). Images were manually inspected for abnormalities and artefacts.

### Brain image preprocessing and analysis

Data were preprocessed and analysed using the CONN-fMRI Functional Connectivity toolbox v20b (Whitfield-Gabrieli and Nieto-Castanon, 2012) (http://www.nitrc.org/projects/conn), MATLAB R2019a (MathWorks, Natick, MA, USA), and SPM12 (The Wellcome Department of Cognitive Neurology, London, UK, http://www.fil.ion.ucl.ac.uk/spm/software/spm12). All sequences were preprocessed using the default pipeline of the CONN toolbox to remove physiological confounds such as head movements: resampling to 2-mm voxels, realigning, unwarping, centring, slice time correction, normalisation using the Montreal Neurological Institute (MNI) echo-planar imaging template, smoothing using an 8-mm Gaussian kernel, and outlier detection. Data were denoised, and signal data from the cerebrospinal fluid and white matter were regressed out and processed with a band-pass filter of 0.008–0.09 Hz to reduce noise effects from physiological sources and scanner drift. Additional image scrubbing, using ART (artefact detection tools) in the CONN toolbox with a frame-wise displacement threshold of 0.5, outlier timepoints at 97^th^ percentiles, global signal Z-scores of 5, and linear de-trending were performed.

### Statistical analysis

ROI-to-ROI maps for the whole brain were constructed using the toolbox. All ROIs were defined using the toolbox, based on the Harvard-Oxford atlas. The mean time series of each seed region was used as a predictor in a multiple regression general linear model at each ROI. An analysis of covariance controlling for age, sex, and IQ was performed between the patients and controls. Graph theory parameters of the whole brain network were calculated using the toolbox with a false discovery rate (FDR) adjusted *p* < 0.05 as the threshold. The following parameters were analysed.

### Degree

Degree refers to the number of edges from/to each node. It represents a measure of network centrality, showing the degree of local connectedness of the ROI (Nieto-Castanon, 2020).

### Betweenness centrality

Betweenness centrality also represents the centrality of a node. It is the proportion of times that a certain node is part of a shortest-path between any two nodes within the graph (Nieto-Castanon, 2020).

### Global efficiency

Global efficiency is the average of inverse-distances between a node and all other nodes in the network. It represents a measure of centrality within the network, showing the degree of global connectedness of the node (Nieto-Castanon, 2020).

### Local efficiency

Local efficiency refers to the global efficiency of a node within the neighbouring sub-graph of the node. It represents a measure of local coherence (Nieto-Castanon, 2020).

ROIs that showed significant differences in the data-driven network analysis were considered to have high/low centrality within the network and were selected as seed areas for a hypothesis-driven ROI-to-ROI resting-state functional connectivity (rsFC) analysis. The threshold for ROI-to-ROI connections was set as uncorrected *p* < 0.05 for assessing statistical significance.

Statistical analyses of clinical and demographic data were performed using SPSS version 23 (IBM Corp., Armonk, NY, USA). The significance level was set at *p* < 0.05. Means and standard deviations for demographic and clinical data were calculated.

## Results

### Demographics and clinical characteristics

Several enrolled participants were excluded from analysis based on our exclusion criteria. Out of the 39 patients one patient was excluded due to an IQ under 70, one patient was excluded due to co-diagnosis of obsessive-compulsive disorder (OCD), two patients were excluded due to imaging artefacts, and 13 patients failed to undergo a full MRI scan due to motion artefacts. Two out of the 37 controls were reassigned to the patient group after clinical assessment, one participant was excluded due to poor image quality, and eight failed to undergo MRI scans. A total of 22 patients (14 males, 4 females) and 26 controls (13 males, 9 females) were included in the final analysis, and 6 patients were co-diagnosed with ADHD. Details are shown in table 1.

**Table 1.** Demographics and clinical measures

	Patients (*n* = 22)	Controls (*n* = 26)	*p*-value
Sex (male/female)	18/4	14/12	
Age	10.36 ± 2.84	10.15 ± 2.15	0.772
IQ	96.45 ± 9.16	102.62 ± 10.05	0.033*
YGTSS	24.73 ± 7.81	0 ± 0	<0.001*
Comorbid ADHD	6	0	

* *p* < 0.05.

### Network analysis between patients and controls

**Figure 1.**
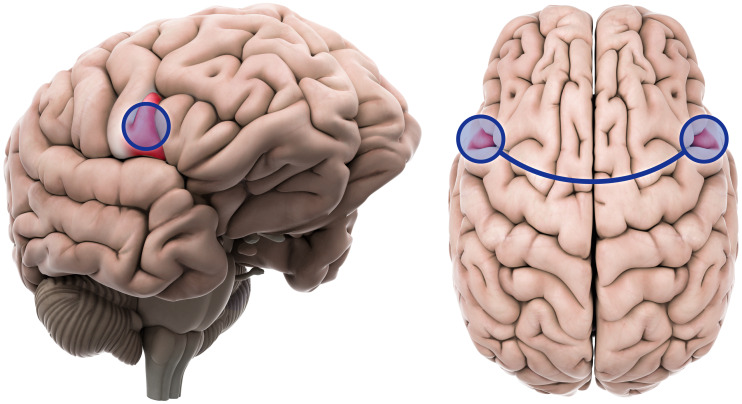
The inferior frontal gyrus pars opercularis showing significantly increased network hubness and resting state functional connectivity in tic disorder patients. Left: inferior frontal gyrus pars opercularis, left. Right: inferior frontal gyrus pars opercularis, both.

All four graph theory measures were analysed for all ROIs. Degree, betweenness centrality, and local efficiency did not show any significant differences between patients and controls. The left inferior frontal gyrus pars opercularis showed significantly higher global efficiency in the patient group (β = 0.06, T = 3.96, *p*-FDR = 0.0428). All graph theory measures did not show significant associations with YGTSS scores or other clinical measures. Table 2 shows statistical details and Fig. 1 shows a graphical representation.

**Table 2.** Network analysis and ROI-to-ROI analysis between patients and controls

ROI	β	T	*p*-FDR	*p*-unc
Global efficiency				
Inferior frontal gyrus pars opercularis, left	0.06	3.96	0.0428	0.0003
ROI-to-ROI				
Inferior frontal gyrus pars opercularis, left to Inferior frontal gyrus pars opercularis, right		2.05	0.9996	0.0464

Abbreviations: ROI, region of interest; *p*-FDR, false discovery rate adjusted *p*-value; p-unc, uncorrected *p*-value.

### ROI-to-ROI analysis between patients and controls

**Figure 2.**
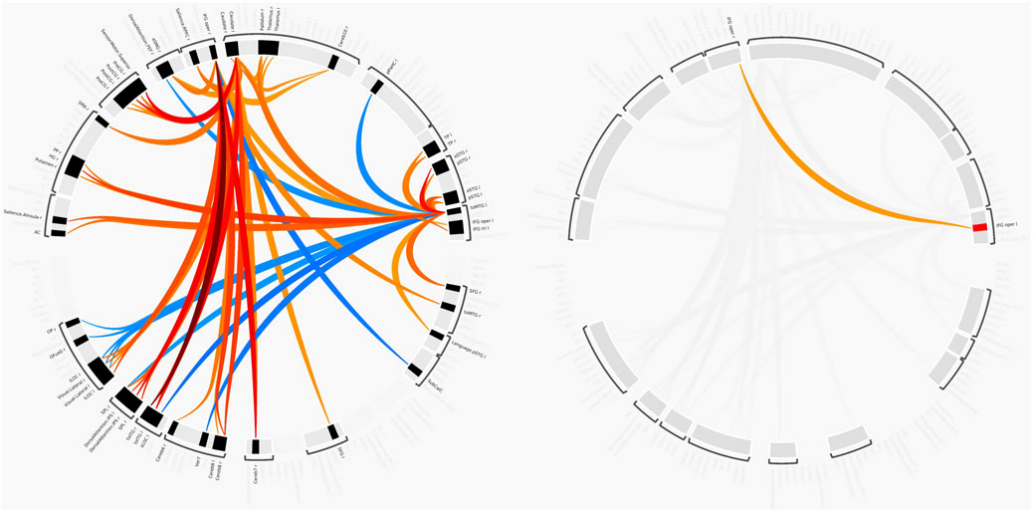
ROI-to-ROI connectome ring. Left: connectome ring of all significant ROI-to-ROI interactions; right: significant connection between left and right inferior frontal gyrus pars opercularis.

Tic disorder patients and healthy controls showed different functional connectivities between various brain areas. ROIs with significant centrality were used as seed ROIs for analysis. The rsFC between the left inferior frontal gyrus pars opercularis and the right inferior frontal gyrus pars opercularis (T = 2.05, *p* = 0.0464) was shown to be higher in the patient group. Table 2 includes detailed statistics on the ROI-to-ROI analysis and Fig. 1 shows a graphical representation. Fig. 2 shows connectome ring representations of the ROI of significant network centrality, as well as the connectome ring of all significant rsFC differences between patients and controls.

## Discussion

One of the strengths of this study is in the participant selection. Although it is not a perfectly homogeneous group, efforts to maintain the highest possible level of homogeneity aimed to minimise the variability typically seen in more diverse groups. This approach ensures that the observed neuroimaging patterns are more confidently attributed to tic disorders rather than confounded by other co-existing psychiatric conditions. It is already widely known that a notable subset of tic disorder patients is co-diagnosed with OCD. Decades of research have been invested on this topic, and even though a clear explanation has yet to be provided, we are aware that tic disorder patients, OCD patients, and patients with both disorders show many significant differences (Coffey *et al*., 1998; Lebowitz *et al*., 2012). We therefore decided to only include patients without OCD to maximise homogeneity.

Our analysis revealed heightened network centrality within the left inferior frontal gyrus pars opercularis. This elevated centrality indicates that this region exhibits increased hubness in patients with tic disorders, suggesting its enhanced significance within their neural network. The pars opercularis is the part of the inferior frontal gyrus that is posterior to the ascending ramus of the lateral sulcus, overlying the insular cortex. This region was known to associate with recognising spoken language as it overlaps with a segment of Broca’s area (Schremm *et al*., 2018) and was also believed to play a central role in executive control and cognitive inhibition (Aron *et al*., 2004). Recent advancements in neuroimaging have underscored the significance of this region in inhibitory controls (Swick *et al*., 2008). Notably, lesions in the right inferior frontal gyrus have been correlated with deficits in stop-signal inhibition (Aron *et al*., 2003). A non-invasive study employing theta burst transcranial magnetic stimulation on this region also revealed augmented inhibitory control in patients with nicotine addiction (Newman-Norlund *et al*., 2020). Based on these and other related studies, it has been concluded that this region is posited to have a unique association with motor inhibition. Interestingly, the inhibitory function of the right inferior frontal gyrus appears to manifest predominantly in later stages of life, while the left inferior frontal gyrus demonstrates heightened inhibitory activity in children (Bunge *et al*., 2002). Similar to its right counterpart, lesions of the left inferior frontal gyrus were also linked to deficits in GoNoGo tasks (Swick *et al*., 2008). Altogether, these findings highlight the critical role of both right and left inferior frontal gyri in response inhibition, a hallmark characteristic that is affected in tic disorder. A recent study observing increased surface curvature of the pars opercularis and the pars triangularis in tic disorder patients compared to controls further substantiates this assertion (McCann *et al*., 2022). The authors also suggested that abnormalities of this region are possibly associated with vocal tics, since damage to this area may give rise to hesitant speech that is also evident in Broca’s aphasia, but it has to be noted that the brain regions responsible for language and inhibition do not completely overlap, and there is still a lack of research supporting the idea that regions associated with language also play a role in inhibition. A diffusion tensor imaging (DTI) study on adults with tic disorder showed decreased fractional anisotropy of several brain regions including the left pars opercularis, further suggesting that alterations of this area are related to the inhibitory impairments in tic disorder (Müller-Vahl *et al*., 2014). This was also demonstrated in a study that observed impaired ability to stop an action while the right pars opercularis was temporarily deactivated by repetitive transcranial magnetic stimulation (Chambers *et al*., 2006). Collectively, these findings suggest the pars opercularis’s involvement in the inhibitory mechanism governing tics. Increased hubness of this area can be hypothesised to be an acquired change in response to frequent motor tics during the developmental ages. This ‘secondary neuroplastic compensatory mechanism’ in tic disorders was already proposed by many researchers including Muller-Vahl and others (Müller-Vahl *et al*., 2014). Such compensatory adaptations are thought to result from continuous tic suppression, also possibly linked to the known decline of tic symptoms with increasing age (Peterson *et al*., 2001).

A study by Openneer *et al.*, also investigated the brain network of tic disorder by comparing patients with tic disorder, ADHD, and healthy controls (Openneer *et al*., 2020). Patients were also divided into groups with and without a co-diagnosis of ADHD. The authors reported that tic disorder patients with ADHD showed lower local efficiency and clustering coefficients in the default-mode network compared to controls, and in the fronto-parietal network when compared to ADHD patients. The pars opercularis, which was significant in our study, does not traditionally belong to one of those networks, so our results do not exactly match those of Openneer *et al*., Our results show increased network centrality in tic disorder patients whereas Openneer et al., reported decreased network centrality in the patient group. Of course, it must be noted that the two studies had different patient groups (all tic disorder patients vs. only tic disorder patients with ADHD), but the different results indicate that tic disorder is a complicated mixture of various brain activities.

Our study is not without limitations. First, the small number of enrolled patients is a major limitation of all tic disorder studies. The young age and frequent movement inherent to tic disorder patients make it difficult to obtain high quality MRI data. Nonetheless, it is crucial to highlight the high homogeneity and drug-naïve status of our patient group. Second, the cross-sectional design of the study made it impossible to verify our results through a prospective time period. Third, it can be observed that the YGTSS scores of the enrolled patients are not very high. In addition, to participate in the study, children and adolescents with motor tics had to be able to lie still and cooperate with the MRI for a certain amount of time. This was a limiting factor for participants with severe, uncontrolled tics. Therefore, some participants with severe tics were not able to participate in the study. This contributed to lower YGTSS scores in those who were able to participate. Fourth, there is a gender imbalance in the study population. While it is expected that more males are affected due to the nature of the disorder, it is undeniable that a more balanced sample would have been more appropriate for the study.

We did not separate patients with comorbid ADHD. Although some previous studies suggested that tic disorders with comorbid ADHD may have different pathophysiological mechanisms compared to those without ADHD, these patients were not excluded due to practical constraints in the study design. In future research, we aim to address this by using a larger sample size to enable such a distinction. Nevertheless, our study suggests that the brains of tic disorder patients may exhibit different developmental traces when compared to controls, as they are continuously inhibiting tics during their developmental ages. These findings will aid in unveiling the pathophysiology of tic disorders. Further research and follow-up studies are necessary to verify our results and to develop clinical applications.

## Conclusion

This study revealed that tic disorder patients have increased network hubness in the left inferior frontal gyrus pars opercularis. This region also showed higher rsFC with its right counterpart in the patient group relative to controls. This area is considered to be linked with inhibitory mechanisms, and its increased hubness could be associated to the constant suppression of frequent tics during developmental years.

## Data Availability

Please contact the corresponding author for data and material.
